# *fac*-Tris(dimethyl sulfoxide-κ*O*)tris­(thio­cyanato-κ*N*)iron(III)

**DOI:** 10.1107/S2414314626005924

**Published:** 2026-06-12

**Authors:** Mohamed Abdellatif Bensegueni, Aouatef Cherouana

**Affiliations:** a Unité de recherche de chimie de l’environnement et moléculaire structurale, Université Constantine 1, Frères Mentouri, Constantine, 25000, Algeria; Vienna University of Technology, Austria

**Keywords:** crystal structure, hydrogen bonding, iron complex, iso­thio­cyanate

## Abstract

The title complex, [Fe(SCN)_3_(C_2_H_6_OS)_3_], was obtained under solvothermal conditions. The asymmetric unit contains one neutral mol­ecule in which the central Fe^III^ atom exhibits a distorted octa­hedral coordination environment. The three N-bonded thio­cyanato ligands and the three O-bonded dimethyl sulfoxide ligands adopt a *fac* configuration. In the crystal, weak C—H⋯S hydrogen bonds link the complexes into centrosymmetric dimers that are arranged in layers parallel to (001).

## Abstract 2

The title complex adopts a *fac*-octa­hedral coordination environment around the central Fe^III^ atom with three O-bonded dimethyl sulfoxide ligands and three N-bonded iso­thio­cyanate ligands.

## Graphical abstract

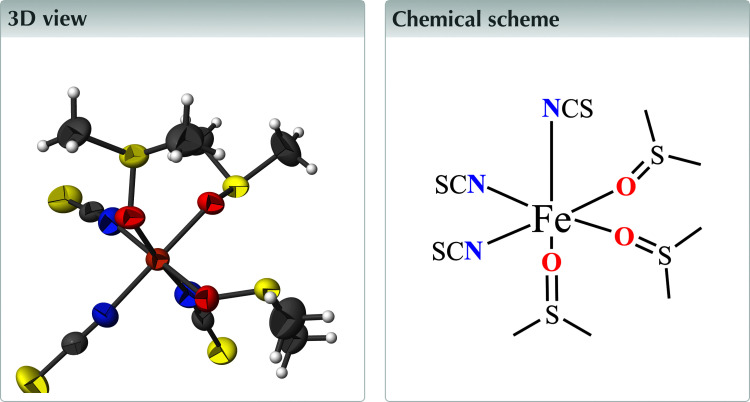

## Structure description

The reaction that led to the serendipitous crystallization of the title complex, Fe(NCS)_3_(DMSO)_3_ (DMSO is di­methyl­sulfoxide), (I), was originally designed for the solvothermal synthesis of a heteroleptic iron complex, with aceto­nitrile serving both as solvent and nitrile substrate for a possible *in situ* azide–nitrile cyclo­addition leading to a tetra­zole-containing ligand.

**Figure 1:**
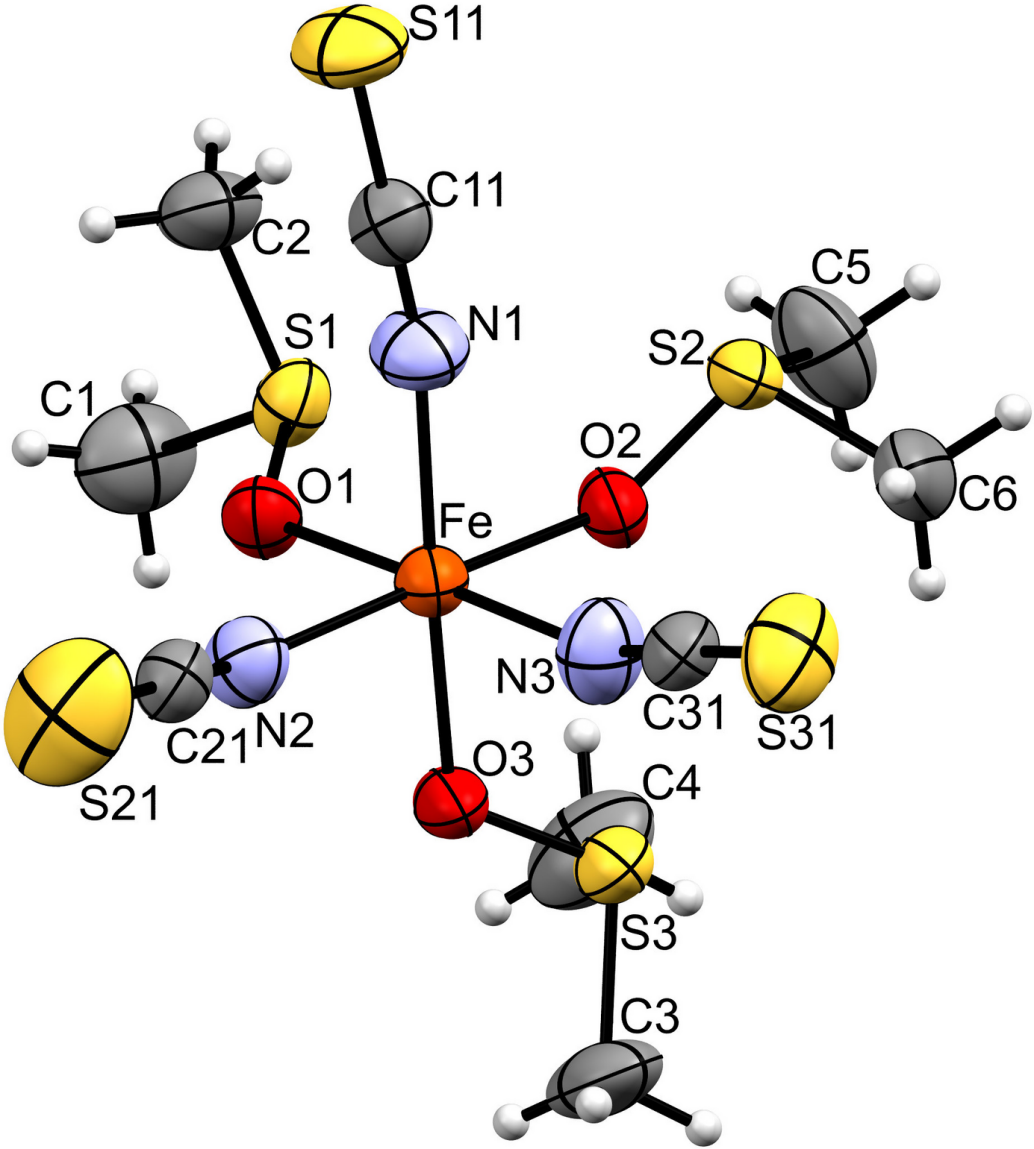
The mol­ecular structure of the title compound with displacement ellipsoids drawn at the 50% probability level.

The asymmetric unit of (I) contains one neutral complex (Fig. 1 ▸). The Fe^III^ atom is in a distorted octa­hedral environment, coordinated by three N atoms from thio­cyanato ligands (N1, N2, N3) and three O atoms from DMSO ligands (O1, O2, O3). The O-bound coordination mode of DMSO is well documented for metal cations classified as ‘hard’ according to the Pearson (1963 ▸) concept, such as Fe^III^. The Fe—N distances between 1.997 (4) and 2.035 (4) Å and the Fe—O distances between 2.031 (3) and 2.043 (3) Å (Table 1 ▸) are consistent with analogous iron(III) iso­thio­cyanate complexes reported by Wang *et al.* (2003 ▸). The configuration around the Fe^III^ atom is *fac*, resulting from the three N-bonded thio­cyanato ligands and the three O-bonded DMSO ligands occupying opposite triangular faces of the octa­hedron. The thio­cyanato ligands are slightly bent, with Fe—N—C angles between 155.5 (4) and 170.6 (4)° and nearly linear N≡C—S angles between 177.3 (4) and 179.3 (4)°. The methyl groups of each of the three DMSO ligands are in an eclipsed conformation relative to each other.

**Figure 2:**
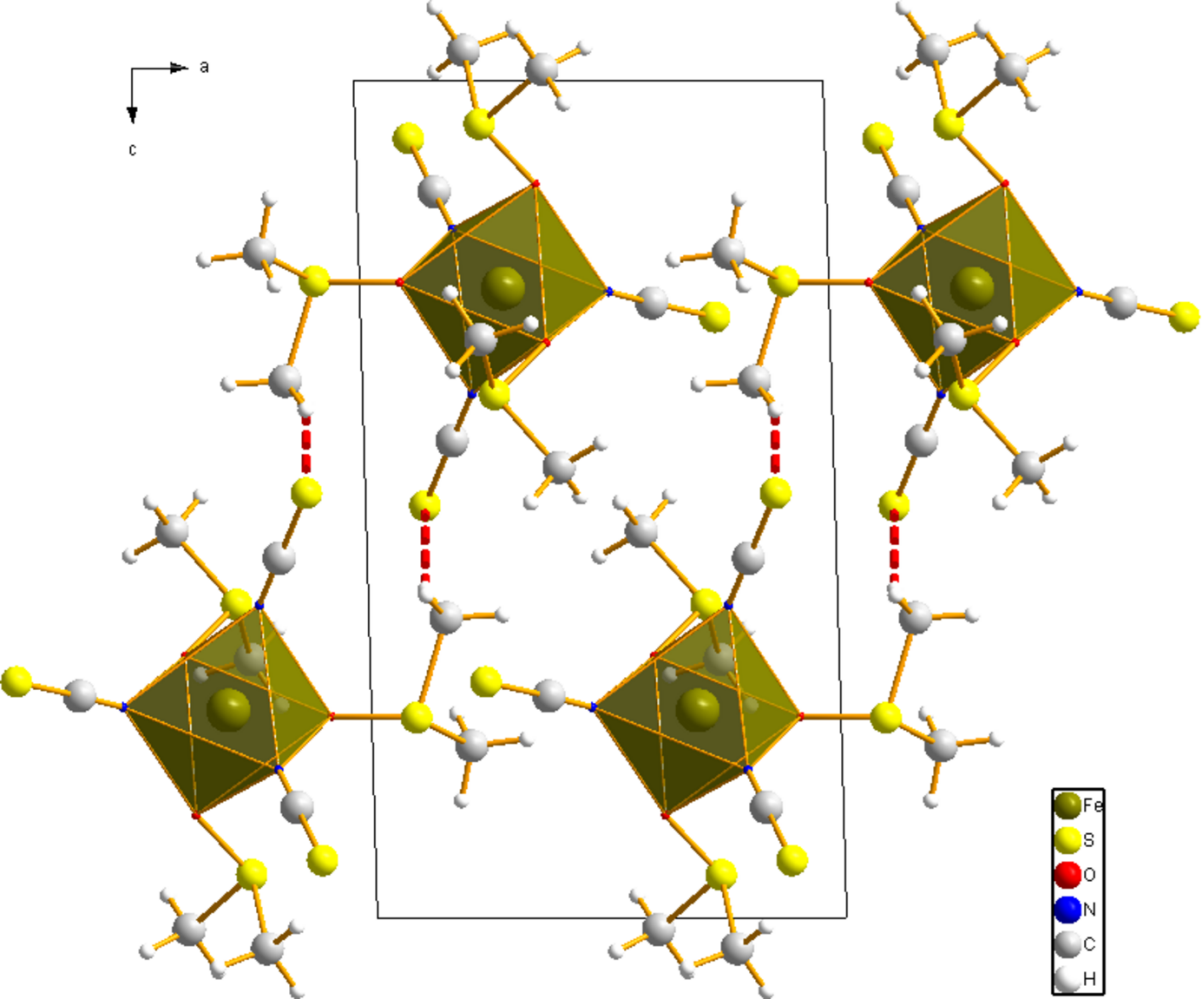
Crystal packing of the title compound in a projection along [010]. The coordination environment around the Fe^III^ atoms is shown in polyhedral representation; red dashed lines indicate weak C—H⋯S hydrogen bonds.

In the crystal of (I), individual mol­ecules are linked by weak C—H⋯S hydrogen bonds (Table 2 ▸) into centrosymmetric dimers that are arranged in layers parallel to (001) (Fig. 2 ▸).

Several crystal structures of iron(III) thio­cyanate complexes and related metal complexes containing oxygen-donor co-ligands have been reported over the past decades. The title complex is most closely related to the *fac*-tris­(dimethyl sulfoxide)(thio­cyanato)­scandium(III) complex crystallizing in the ortho­rhom­bic space group *Pna*2_1_, *Z* = 4, *a* = 14.583 (2), *b* = 14.728 (2), *c* = 9.849 (2) Å, *V* = 2115.4 (6) Å^3^ (Chenskaya *et al.*, 2000 ▸). Both structures comprise mononuclear octa­hedral complexes featuring three N-bonded thio­cyanato ligands and three O-bonded dimethyl sulfoxide ligands with only minor differences in bond lengths reflecting the different nature of the central metal ion. Other related complexes include thio­cyanate/DMSO-containing lanthanide compounds (Bu *et al.*, 2002 ▸; Li *et al.*, 2004 ▸; Miranda *et al.*, 2004 ▸; Ilichev *et al.*, 2023 ▸). However, to the best of our knowledge, no mononuclear iron(III) complex containing both N-bonded thio­cyanato ligands and O-bonded dimethyl sulfoxide ligands has been reported to date.

## Synthesis and crystallization

Potassium thio­cyanate (2 mmol, 0.199 g) and iron(II) sulfate hepta­hydrate (1 mmol, 0.278 g) were dissolved in dimethyl sulfoxide (10 ml) in the presence of ascorbic acid as a reducing agent, and the mixture was stirred for 20 min at room temperature. Aceto­nitrile was present in the reaction medium as the nitrile source. Sodium azide (0.5 mmol, 0.033 g) was dissolved separately in a minimum volume of distilled water and added to the above solution. The reaction mixture was transferred into a 23 ml PTFE-lined stainless-steel autoclave, sealed, and heated at 393 K for 72 h, then allowed to cool slowly to room temperature. Orange prismatic crystals of the title compound were collected by filtration, washed with cold DMSO, and air-dried.

## Refinement

Crystal data, data collection, and structure refinement details are summarized in Table 3 ▸.

## Supplementary Material

Crystal structure: contains datablock(s) I. DOI: 10.1107/S2414314626005924/wm4250sup1.cif

Structure factors: contains datablock(s) I. DOI: 10.1107/S2414314626005924/wm4250Isup2.hkl

CCDC reference: 2541206

Additional supporting information:  crystallographic information; 3D view; checkCIF report

## Figures and Tables

**Table 1:** Selected geometric parameters (Å, °)

Fe—O3	2.031 (3)	Fe—O2	2.041 (3)
Fe—N1	2.035 (4)	Fe—N3	2.016 (4)
Fe—O1	2.043 (3)	Fe—N2	1.997 (4)
			
O3—Fe—N1	174.70 (13)	O2—Fe—O1	88.64 (11)
O3—Fe—O1	87.64 (12)	N3—Fe—O3	90.20 (15)
O3—Fe—O2	85.20 (11)	N3—Fe—O1	177.81 (14)
N1—Fe—O1	89.85 (14)	N2—Fe—O2	175.50 (13)
N1—Fe—O2	90.08 (13)	N2—Fe—N3	92.13 (15)

**Table 2:** Hydrogen-bond geometry (Å, °)

*D*—H⋯*A*	*D*—H	H⋯*A*	*D*⋯*A*	*D*—H⋯*A*
C6—H6*A*⋯S31^i^	0.96	2.84	3.751 (6)	159

Symmetry code: (i) 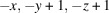.

**Table 3:** Experimental details

Crystal data	
Chemical formula	[Fe(SCN)_3_(C_2_H_6_OS)_3_]
*M*_r_	464.47
Crystal system, space group	Triclinic, *P* 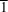
Temperature (K)	150
*a*, *b*, *c* (Å)	7.980 (4), 9.001 (6), 14.340 (7)
α, β, γ (°)	82.233 (18), 87.868 (17), 86.16 (3)
*V* (Å^3^)	1017.8 (9)
*Z*	2
Radiation type	Mo *K*α
μ (mm^−1^)	1.37
Crystal size (mm)	0.20 × 0.15 × 0.10

Data collection	
Diffractometer	Bruker APEXII CCD
Absorption correction	Multi-scan (*SADABS*; Krause *et al.*, 2015 ▸)
*T*_min_, *T*_max_	0.632, 0.746
No. of measured, independent and observed [*I* > 2σ(*I*)] reflections	6890, 3801, 2424
*R*_int_	0.034
(sin θ/λ)_max_ (Å^−1^)	0.610

Refinement	
*R*[*F*^2^ > 2σ(*F*^2^)], *wR*(*F*^2^), *S*	0.044, 0.115, 0.98
No. of reflections	3801
No. of parameters	205
H-atom treatment	H-atom parameters constrained
Δρ_max_, Δρ_min_ (e Å^−3^)	0.35, −0.36

Computer programs: *SMART* and, *SAINT* (Bruker, 2009 ▸), *SHELXS* (Sheldrick, 2008 ▸), *SHELXL* (Sheldrick, 2015 ▸), *OLEX2* (Dolomanov *et al.*, 2009 ▸) and *DIAMOND* (Putz & Brandenburg, 2010 ▸), *publCIF* (Westrip, 2010 ▸).

## full crystallographic data

### fac-Tris(dimethyl sulfoxide-κO)tris(thiocyanato-κN)iron(III) Crystal data

**Table:**

[Fe(SCN)_3_(C_2_H_6_OS)_3_]	*Z* = 2
*M**_r_* = 464.47	*F*(000) = 478
Triclinic, *P*1	*D*_x_ = 1.516 Mg m^−^^3^
*a* = 7.980 (4) Å	Mo *K*α radiation, λ = 0.71073 Å
*b* = 9.001 (6) Å	Cell parameters from 295 reflections
*c* = 14.340 (7) Å	θ = 2.9–22.3°
α = 82.233 (18)°	µ = 1.37 mm^−^^1^
β = 87.868 (17)°	*T* = 150 K
γ = 86.16 (3)°	Prism, orange
*V* = 1017.8 (9) Å^3^	0.20 × 0.15 × 0.10 mm

### fac-Tris(dimethyl sulfoxide-κO)tris(thiocyanato-κN)iron(III) Data collection

**Table:**

Bruker APEXII CCD diffractometer	3801 independent reflections
Radiation source: sealed tube	2424 reflections with *I* > 2σ(*I*)
Graphite monochromator	*R*_int_ = 0.034
Detector resolution: 8 pixels mm^-1^	θ_max_ = 25.7°, θ_min_ = 3.5°
φ and ω scans	*h* = −7→9
Absorption correction: multi-scan (SADABS; Krause *et al.*, 2015)	*k* = −10→10
*T*_min_ = 0.632, *T*_max_ = 0.746	*l* = −17→17
6890 measured reflections	

### fac-Tris(dimethyl sulfoxide-κO)tris(thiocyanato-κN)iron(III) Refinement

**Table:**

Refinement on *F*^2^	Primary atom site location: structure-invariant direct methods
Least-squares matrix: full	Hydrogen site location: inferred from neighbouring sites
*R*[*F*^2^ > 2σ(*F*^2^)] = 0.044	H-atom parameters constrained
*wR*(*F*^2^) = 0.115	*w* = 1/[\s^2^(*F*_o_^2^) + (0.0546*P*)^2^] where *P* = (*F*_o_^2^ + 2*F*_c_^2^)/3
*S* = 0.98	(Δ/σ)_max_ < 0.001
3801 reflections	Δρ_max_ = 0.35 e Å^−^^3^
205 parameters	Δρ_min_ = −0.35 e Å^−^^3^
0 restraints	

### fac-Tris(dimethyl sulfoxide-κO)tris(thiocyanato-κN)iron(III) Special details

**Table:**

Experimental. Single crystals were obtained by solvothermal synthesis at 393 K for 72 h in a PTFE-lined stainless steel autoclave. Reflections 0 0 1, 0 1 0 and 1 1 1 were affected by the beamstop and have been excluded from refinement using OMIT instructions in SHELXL. Data were truncated to 0.82 Ang resolution (SHEL 50 0.82 instruction) to improve data quality and reduce noise at high theta angles.
Geometry. All esds (except the esd in the dihedral angle between two l.s. planes) are estimated using the full covariance matrix. The cell esds are taken into account individually in the estimation of esds in distances, angles and torsion angles; correlations between esds in cell parameters are only used when they are defined by crystal symmetry. An approximate (isotropic) treatment of cell esds is used for estimating esds involving l.s. planes.
Refinement. Refinement of F^2^ against ALL reflections. The weighted R-factor wR and goodness of fit S are based on F^2^, conventional R-factors R are based on F, with F set to zero for negative F^2^. The threshold expression of F^2^ > 2\s(F^2^) is used only for calculating R-factors(gt) etc. and is not relevant to the choice of reflections for refinement. R-factors based on F^2^ are statistically about twice as large as those based on F, and R-factors based on ALL data will be even larger. Reflections 0 0 1, 0 1 0 and 1 1 1 were excluded from the refinement as they were affected by the beamstop (OMIT instructions in SHELXL). Data were truncated to d_min = 0.82 Ang (SHEL 50 0.82 instruction) to improve data quality and reduce noise at high theta angles.

### fac-Tris(dimethyl sulfoxide-κO)tris(thiocyanato-κN)iron(III) Fractional atomic coordinates and isotropic or equivalent isotropic displacement parameters (Å^2^)

**Table:**

	*x*	*y*	*z*	*U*_iso_*/*U*_eq_	
Fe	0.30529 (6)	0.47954 (6)	0.24808 (4)	0.03937 (18)	
S1	0.26516 (13)	0.69120 (12)	0.05193 (7)	0.0482 (3)	
O3	0.3939 (3)	0.6441 (3)	0.31306 (19)	0.0492 (7)	
S3	0.28122 (13)	0.73722 (13)	0.37470 (8)	0.0525 (3)	
S2	−0.09552 (12)	0.56090 (13)	0.23945 (8)	0.0491 (3)	
N1	0.2014 (4)	0.3294 (4)	0.1769 (3)	0.0585 (10)	
O1	0.3801 (3)	0.5967 (3)	0.12312 (17)	0.0473 (7)	
O2	0.0853 (3)	0.6094 (3)	0.24130 (18)	0.0454 (7)	
N3	0.2331 (5)	0.3708 (5)	0.3739 (3)	0.0644 (11)	
C1	0.4020 (7)	0.8140 (6)	−0.0133 (3)	0.0829 (17)	
H1A	0.4454	0.8779	0.0273	0.124*	
H1B	0.3420	0.8744	−0.0631	0.124*	
H1C	0.4933	0.7567	−0.0396	0.124*	
C2	0.2360 (6)	0.5762 (6)	−0.0361 (3)	0.0653 (13)	
H2A	0.3435	0.5402	−0.0593	0.098*	
H2B	0.1756	0.6335	−0.0869	0.098*	
H2C	0.1732	0.4924	−0.0099	0.098*	
C3	0.4139 (7)	0.7773 (7)	0.4617 (3)	0.0900 (19)	
H3A	0.4574	0.6850	0.4966	0.135*	
H3B	0.3515	0.8364	0.5037	0.135*	
H3C	0.5053	0.8322	0.4326	0.135*	
C5	−0.2116 (6)	0.7335 (6)	0.2058 (4)	0.0869 (18)	
H5A	−0.3296	0.7189	0.2141	0.130*	
H5B	−0.1866	0.7684	0.1408	0.130*	
H5C	−0.1815	0.8066	0.2441	0.130*	
C6	−0.1642 (6)	0.5271 (6)	0.3587 (3)	0.0719 (15)	
H6A	−0.1223	0.6008	0.3929	0.108*	
H6B	−0.1230	0.4286	0.3856	0.108*	
H6C	−0.2848	0.5337	0.3623	0.108*	
C31	0.1876 (5)	0.2758 (5)	0.4299 (3)	0.0466 (10)	
C11	0.1646 (5)	0.2381 (5)	0.1325 (3)	0.0473 (10)	
S11	0.11343 (18)	0.11629 (16)	0.06928 (10)	0.0744 (4)	
S31	0.12611 (19)	0.14698 (15)	0.50741 (10)	0.0763 (4)	
C4	0.2545 (8)	0.9126 (6)	0.3089 (4)	0.0949 (19)	
H4A	0.3624	0.9497	0.2904	0.142*	
H4B	0.1938	0.9805	0.3461	0.142*	
H4C	0.1925	0.9050	0.2539	0.142*	
S21	0.7549 (2)	0.12394 (19)	0.28209 (12)	0.1095 (6)	
N2	0.5300 (4)	0.3672 (4)	0.2522 (2)	0.0548 (9)	
C21	0.6214 (5)	0.2630 (5)	0.2652 (3)	0.0473 (10)	

### fac-Tris(dimethyl sulfoxide-κO)tris(thiocyanato-κN)iron(III) Atomic displacement parameters (Å^2^)

**Table:**

	*U*^11^	*U*^22^	*U*^33^	*U*^12^	*U*^13^	*U*^23^
Fe	0.0374 (3)	0.0410 (4)	0.0407 (3)	−0.0035 (2)	−0.0001 (2)	−0.0089 (3)
S1	0.0504 (6)	0.0506 (7)	0.0416 (6)	0.0079 (5)	0.0034 (5)	−0.0064 (5)
O3	0.0383 (15)	0.0578 (18)	0.0571 (17)	−0.0050 (13)	0.0010 (12)	−0.0281 (15)
S3	0.0465 (6)	0.0628 (8)	0.0526 (7)	−0.0003 (5)	−0.0010 (5)	−0.0255 (6)
S2	0.0349 (5)	0.0629 (7)	0.0551 (7)	−0.0055 (5)	−0.0023 (5)	−0.0260 (6)
N1	0.062 (2)	0.049 (2)	0.070 (3)	−0.0081 (19)	−0.0061 (19)	−0.021 (2)
O1	0.0420 (15)	0.0564 (18)	0.0419 (16)	−0.0007 (14)	−0.0024 (12)	−0.0017 (13)
O2	0.0329 (14)	0.0505 (17)	0.0543 (17)	−0.0035 (12)	0.0044 (12)	−0.0135 (14)
N3	0.067 (3)	0.068 (3)	0.053 (2)	−0.002 (2)	0.0080 (19)	0.005 (2)
C1	0.113 (5)	0.065 (4)	0.069 (3)	−0.027 (3)	−0.003 (3)	0.010 (3)
C2	0.075 (3)	0.073 (3)	0.051 (3)	−0.010 (3)	−0.013 (2)	−0.015 (2)
C3	0.092 (4)	0.123 (5)	0.066 (3)	0.014 (4)	−0.026 (3)	−0.053 (3)
C5	0.052 (3)	0.090 (4)	0.111 (5)	0.011 (3)	−0.008 (3)	0.005 (4)
C6	0.049 (3)	0.107 (4)	0.063 (3)	−0.021 (3)	0.007 (2)	−0.019 (3)
C31	0.047 (2)	0.052 (3)	0.043 (2)	−0.003 (2)	0.0030 (19)	−0.015 (2)
C11	0.048 (2)	0.042 (3)	0.051 (3)	0.005 (2)	−0.004 (2)	−0.005 (2)
S11	0.0858 (9)	0.0629 (8)	0.0830 (9)	−0.0047 (7)	−0.0180 (7)	−0.0367 (7)
S31	0.0960 (10)	0.0569 (8)	0.0751 (9)	−0.0222 (7)	0.0257 (7)	−0.0044 (7)
C4	0.139 (6)	0.067 (4)	0.078 (4)	0.020 (4)	−0.005 (4)	−0.021 (3)
S21	0.1331 (15)	0.0751 (11)	0.1091 (13)	0.0568 (11)	−0.0088 (11)	−0.0012 (9)
N2	0.053 (2)	0.055 (2)	0.056 (2)	0.011 (2)	−0.0037 (18)	−0.0123 (19)
C21	0.051 (3)	0.048 (3)	0.043 (2)	−0.002 (2)	0.0039 (19)	−0.010 (2)

### fac-Tris(dimethyl sulfoxide-κO)tris(thiocyanato-κN)iron(III) Geometric parameters (Å, º)

**Table:**

Fe—O3	2.031 (3)	C2—H2A	0.9600
Fe—N1	2.035 (4)	C2—H2B	0.9600
Fe—O1	2.043 (3)	C2—H2C	0.9600
Fe—O2	2.041 (3)	C3—H3A	0.9600
Fe—N3	2.016 (4)	C3—H3B	0.9600
Fe—N2	1.997 (4)	C3—H3C	0.9600
S1—O1	1.529 (3)	C5—H5A	0.9600
S1—C1	1.759 (5)	C5—H5B	0.9600
S1—C2	1.768 (4)	C5—H5C	0.9600
O3—S3	1.527 (3)	C6—H6A	0.9600
S3—C3	1.755 (5)	C6—H6B	0.9600
S3—C4	1.731 (6)	C6—H6C	0.9600
S2—O2	1.538 (3)	C31—S31	1.584 (5)
S2—C5	1.773 (5)	C11—S11	1.596 (5)
S2—C6	1.769 (5)	C4—H4A	0.9600
N1—C11	1.164 (5)	C4—H4B	0.9600
N3—C31	1.158 (5)	C4—H4C	0.9600
C1—H1A	0.9600	S21—C21	1.587 (5)
C1—H1B	0.9600	N2—C21	1.148 (5)
C1—H1C	0.9600		
			
O3—Fe—N1	174.70 (13)	S1—C2—H2A	109.5
O3—Fe—O1	87.64 (12)	S1—C2—H2B	109.5
O3—Fe—O2	85.20 (11)	S1—C2—H2C	109.5
N1—Fe—O1	89.85 (14)	H2A—C2—H2B	109.5
N1—Fe—O2	90.08 (13)	H2A—C2—H2C	109.5
O2—Fe—O1	88.64 (11)	H2B—C2—H2C	109.5
N3—Fe—O3	90.20 (15)	S3—C3—H3A	109.5
N3—Fe—N1	92.26 (17)	S3—C3—H3B	109.5
N3—Fe—O1	177.81 (14)	S3—C3—H3C	109.5
N3—Fe—O2	90.79 (13)	H3A—C3—H3B	109.5
N2—Fe—O3	91.36 (13)	H3A—C3—H3C	109.5
N2—Fe—N1	93.23 (15)	H3B—C3—H3C	109.5
N2—Fe—O1	88.32 (13)	S2—C5—H5A	109.5
N2—Fe—O2	175.50 (13)	S2—C5—H5B	109.5
N2—Fe—N3	92.13 (15)	S2—C5—H5C	109.5
O1—S1—C1	103.3 (2)	H5A—C5—H5B	109.5
O1—S1—C2	105.5 (2)	H5A—C5—H5C	109.5
C1—S1—C2	97.6 (2)	H5B—C5—H5C	109.5
S3—O3—Fe	122.26 (16)	S2—C6—H6A	109.5
O3—S3—C3	104.5 (2)	S2—C6—H6B	109.5
O3—S3—C4	104.9 (2)	S2—C6—H6C	109.5
C4—S3—C3	100.5 (3)	H6A—C6—H6B	109.5
O2—S2—C5	102.7 (2)	H6A—C6—H6C	109.5
O2—S2—C6	105.63 (19)	H6B—C6—H6C	109.5
C6—S2—C5	99.1 (3)	N3—C31—S31	179.3 (4)
C11—N1—Fe	170.6 (4)	N1—C11—S11	178.5 (4)
S1—O1—Fe	125.96 (15)	S3—C4—H4A	109.5
S2—O2—Fe	128.93 (17)	S3—C4—H4B	109.5
C31—N3—Fe	160.1 (4)	S3—C4—H4C	109.5
S1—C1—H1A	109.5	H4A—C4—H4B	109.5
S1—C1—H1B	109.5	H4A—C4—H4C	109.5
S1—C1—H1C	109.5	H4B—C4—H4C	109.5
H1A—C1—H1B	109.5	C21—N2—Fe	155.5 (4)
H1A—C1—H1C	109.5	N2—C21—S21	177.3 (4)
H1B—C1—H1C	109.5		
			
Fe—O3—S3—C3	147.3 (3)	C2—S1—O1—Fe	−99.5 (2)
Fe—O3—S3—C4	−107.4 (3)	C5—S2—O2—Fe	−166.8 (2)
C1—S1—O1—Fe	158.6 (2)	C6—S2—O2—Fe	89.7 (3)

### fac-Tris(dimethyl sulfoxide-κO)tris(thiocyanato-κN)iron(III) Hydrogen-bond geometry (Å, º)

**Table:**

*D*—H···*A*	*D*—H	H···*A*	*D*···*A*	*D*—H···*A*
C6—H6*A*···S31^i^	0.96	2.84	3.751 (6)	159

Symmetry code: (i) −*x*, −*y*+1, −*z*+1.

## References

[bb1] Bruker. (2009). *SMART* and *SAINT*. Bruker AXS Inc., Madison, Wisconsin, USA.

[bb2] Bu, X.-H., Weng, W., Li, J.-R., Chen, W. & Zhang, R.-H. (2002). *Inorg. Chem.* **41**, 413–415. 10.1021/ic010605b 11800632

[bb3] Chenskaya, V., Virovets, A. V., Gromilov, S. A., Podberezskaya, N. V. & Cherkasova, T. G. (2000). *Inorg. Chem. Commun.* **3**, 482–485.

[bb4] Dolomanov, O. V., Bourhis, L. J., Gildea, R. J., Howard, J. A. K. & Puschmann, H. (2009). *J. Appl. Cryst.* **42**, 339–341.

[bb5] Ilichev, V. A., Rogozhin, A. F., Rumyantcev, R. V., Kozlova, E. A., Fukin, G. K., Yablonskiy, A. N., Andreev, B. A. & Bochkarev, M. N. (2023). *Inorg. Chem.* **62**, 12625–12629. 10.1021/acs.inorgchem.3c01349 37523240

[bb6] Krause, L., Herbst-Irmer, R., Sheldrick, G. M. & Stalke, D. (2015). *J. Appl. Cryst.* **48**, 3–10. 10.1107/S1600576714022985 PMC4453166 26089746

[bb7] Li, J.-R., Bu, X.-H. & Zhang, R.-H. (2004). *Inorg. Chem.* **43**, 237–244. 10.1021/ic034772i 14704073

[bb8] Miranda, P., Zukerman-Schpector, J., Serrano, P. C., Vicentini, G. & Zinner, L. B. (2004). *J. Alloys Compd.* **374**, 358–361.

[bb9] Pearson, R. G. (1963). *J. Am. Chem. Soc.* **85**, 3533–3539.

[bb10] Putz, H. & Brandenburg, K. (2010). *DIAMOND*. Crystal Impact, Bonn, Germany.

[bb11] Sheldrick, G. M. (2008). *Acta Cryst.* A**64**, 112–122. 10.1107/S0108767307043930 18156677

[bb12] Sheldrick, G. M. (2015). *Acta Cryst.* C**71**, 3–8.

[bb13] Wang, M.-S., Cai, L.-Z., Guo, G.-C. & Huang, J.-S. (2003). *Acta Cryst.* E**59**, m436–m437.

[bb14] Westrip, S. P. (2010). *J. Appl. Cryst.* **43**, 920–925.

